# Supplementary material for: Investigation of Drosophila fruitless neurons that express Dpr/DIP cell adhesion molecules
Source: eLife. 2021 Feb 22;10:e63101. doi: 10.7554/eLife.63101 (PMC7972454; doi:10.7554/eLife.63101)
Supplement: Figure 1—source data 1. — This is an excel table that contains the number of times a FruM binding site is found in either a dpr or DIP gene. [file elife-63101-fig1-data1.docx]

| **Number of Fru^M^ binding motifs in each of the *dprs*/*DIPs*** | | | | | | | |
| --- | --- | --- | --- | --- | --- | --- | --- |
| Symbol | Fru^MA^ | Fru^MB^ | Fru^MC^ | Symbol | Fru^MA^ | Fru^MB^ | Fru^MC^ |
| *dpr1* | 15 | 1 | 1 | *dpr17* | 3 | 1 | 0 |
| *dpr2* | 4 | 2 | 2 | *dpr18* | 3 | 1 | 0 |
| *dpr3* | 10 | 4 | 1 | *dpr19* | 1 | 3 | 0 |
| *dpr4* | 2 | 0 | 0 | *dpr20* | 0 | 0 | 0 |
| *dpr5* | 3 | 2 | 0 | *dpr21* | 19 | 7 | 6 |
| *dpr6* | 17 | 3 | 0 | *DIP-α* | 4 | 1 | 0 |
| *dpr7* | 2 | 1 | 0 | *DIP-β* | 6 | 2 | 3 |
| *dpr8* | 19 | 3 | 1 | *DIP-γ* | 6 | 3 | 1 |
| *dpr9* | 4 | 2 | 1 | *DIP-δ* | 3 | 1 | 0 |
| *dpr10* | 7 | 3 | 0 | *DIP-ε* | 5 | 0 | 1 |
| *dpr11* | 9 | 1 | 2 | *DIP-ζ* | 2 | 0 | 0 |
| *dpr12* | 5 | 0 | 0 | *DIP-η* | 3 | 0 | 0 |
| *dpr13* | 4 | 0 | 2 | *DIP-θ* | 8 | 1 | 0 |
| *dpr14* | 0 | 0 | 0 | *DIP-ι* | 2 | 0 | 0 |
| *dpr15* | 8 | 2 | 0 | *DIP-κ* | 9 | 1 | 1 |
| *dpr16* | 2 | 1 | 1 | *DIP-λ* | 38 | 5 | 5 |
| Number of motifs identified in (Dalton *et al.* 2013) | | | | | | | |
